# 超高效液相色谱-串联质谱法测定动物肌肉组织中四环素类抗生素

**DOI:** 10.3724/SP.J.1123.2025.03014

**Published:** 2025-12-08

**Authors:** Kun CHU, Jinhua LI, Qianqian WANG, Xiaotong LI, Shuai WU, Chen CHEN

**Affiliations:** 1.烟台市食品药品检验检测中心，山东 烟台 264000; 1. Yantai Testing Center for Food and Drug，Yantai 264000，China; 2.中国科学院烟台海岸带研究所，山东 烟台 264003; 2. Yantai Institute of Coastal Zone Research，Chinese Academy of Sciences，Yantai 264003，China

**Keywords:** 四环素类抗生素, 固相萃取, 超高效液相色谱-串联质谱, 动物肌肉组织, tetracyclines （TCs）, solid-phase extraction （SPE）, ultra performance liquid chromatography-tandem mass spectrometry （UPLC-MS/MS）, animal muscle tissue

## Abstract

四环素类抗生素（tetracyclines， TCs）是一类广谱抗生素，广泛应用于畜禽及水产养殖业。TCs在养殖过程中存在滥用现象，导致其在养殖动物体内残留，对人类健康构成潜在风险。本研究基于超高效液相色谱-串联质谱（UPLC-MS/MS）技术，建立了动物肌肉组织样品（猪肉、鸡肉和鱼肉）中TCs残留量的检测方法，并对样品前处理方法和色谱、质谱条件进行了研究优化。称取2 g均质后的动物肌肉组织样品，加入10 mL含0.2%甲酸的80%乙腈水溶液，振荡提取，低温离心。上清液使用Oasis PRiME HLB固相萃取柱快速一步式除杂净化，净化液经氮气吹干复溶后，进行UPLC-MS/MS分析。待测分析物经Eclipse Plus C_18_色谱柱（100 mm×2.1 mm，3.5 μm）梯度分离洗脱，在正离子电喷雾（ESI^+^）、多反应监测（MRM）模式下扫描，采用基质曲线外标法定量。结果表明，TCs在1～500 ng/mL范围内具有良好的线性关系，相关系数（*r* ^2^）均大于0.994，检出限为0.10～0.15 μg/kg，定量限为0.20～0.50 μg/kg。TCs在低、中、高3个水平（1、5、10 μg/kg）下的平均加标回收率为62.6%～119.0%，相对标准偏差（RSD）为2.0%～9.8％（*n*＝7）。采用本方法检测60份动物肌肉组织样品，TCs的检出率分别为10%、15%和5%。本方法易于操作，具有较高的准确度和精密度，适用于动物肌肉组织中TCs残留量的常规检测。

## Abstract (en)

Tetracyclines （TCs） are broad-spectrum antibiotics that are classified as natural or semi-synthetic. Natural TCs include chlortetracycline， tetracycline， and oxytetracycline， while semi-synthetic ones include doxycycline， minocycline， methacycline， and demeclocycline， among others. While TCs are widely used in the livestock， poultry， and aquaculture industries， their indiscriminate use detrimentally affects ecosystems， and residual TCs in animals can adversely affect human health. In this study， we developed an ultra-performance liquid chromatography-tandem mass spectrometry （UPLC-MS/MS） method for determining TCs， including minocycline， 4-epioxytetracycline， 4-epitetracycline， oxytetracycline， tetracycline， demeclocycline， 4-epichlortetracycline， chlortetracycline， methacycline， and doxycycline. The developed protocol was used to establish a method for qualitatively and quantitatively analyzing TCs in animal muscle tissue （pork， chicken， and fish meat）. To this end， we systematically optimized the mass spectrometry （MS） parameters， solid-phase extraction （SPE） cartridge， and extraction conditions of the method. Animal muscle-tissue samples were homogenized and extracted with 10 mL of 80% acetonitrile aqueous solution containing 0.2% formic acid. How the acetonitrile/formic acid ratio affects the TC-extraction efficiency was investigated using one-way analysis. The supernatant was purified using an Oasis PRiME HLB solid-phase-extraction cartridge， evaporated under flowing nitrogen， and redissolved. Two different C_18_ UPLC columns were systematically evaluated， and the optimal UPLC conditions were established for the TCs. The Eclipse Plus C_18_ column （100 mm×2.1 mm， 3.5 μm） was used for separation. The effects of mobile phases A （0.1% formic acid aqueous solution， 5 mmol/L ammonium acetate aqueous solution， and 5 mmol/L ammonium formate aqueous solution） and B （methanol or acetonitrile） on the separation and response values of the TCs were investigated. Optimal response values and peak shapes were obtained using 0.1% formic acid aqueous solution as mobile phase A and 0.1% formic acid acetonitrile solution as mobile phase B， at a flow rate of 0.2 mL/min and a sample injection volume of 5 μL. Gradient elution was performed as follows： 0–2.0 min， 5%B； 2.0–3.5 min， 5%B-15%B； 3.5–7.0 min， 15%B-20%B； 7.0–9.0 min， 20%B-65%B； 9.0–9.1 min， 65%B-90%B； 9.1–10.0 min， 90%B； 10.0–10.1 min， 90%B-5%B； 10.1–12 min， 5%B. The effect of the glass sample bottle on adsorption was also investigated. Both positive- and negative-ion modes were explored in the UPLC-MS/MS experiment to fully scan the parent ions. Positive mode was selected for electrospray ionization （ESI）. Two product ions that exhibit strong signals and minimal interference were selected for quantitative and qualitative ion analyses， with quantification performed using the external standard method. Tandem mass spectrometry （MS/MS） was performed in positive electrospray ionization （ESI^+^） and multiple reaction monitoring （MRM） modes. The following MS/MS parameters were used： capillary voltage， 0.5 kV； cone voltage （CV）， 30 V； ion-source temperature， 150 ℃； desolvation temperature， 300 ℃； desolvation gas flow， 800 L/h. Other instrument settings， such as the collision energy （CE） and collision gas flow， were also optimized. The TCs exhibited good linearities within the 1–500 ng/mL mass-concentration range， with all correlation coefficients （*r* ^2^） above 0.994， and limits of detection and quantification （LODs and LOQs） of 0.10–0.15 and 0.20–0.50 μg/kg， respectively. The target analytes exhibited average recoveries of between 62.6% and 119.0% at three levels （1.0， 5.0， and 10.0 μg/kg）， with relative standard deviations （RSDs， *n*=7） ranging from 2.0% to 9.8%. The developed method was used to determine TCs in 60 animal muscle-tissue samples acquired from a fresh-food supermarket. Tetracycline was detected at a rate of 10% in 20 pork samples， while doxycycline was detected at a rate of 15% in 20 chicken samples， and oxytetracycline was detected at a rate of 5% in 20 fish-meat samples. Minocycline， 4-epioxytetracycline， 4-epitetracycline， tetracycline， demeclocycline， 4-epichlortetracycline， chlortetracycline， and methacycline were not detected. The developed method is simple to operate， highly sensitive， and can be used to precisely and accurately determine TCs. Accordingly， it is suitable for determining the abovementioned ten tetracycline antibiotics in animal muscle tissue.

四环素类抗生素（tetracyclines，TCs）是由放线菌产生的四并苯化合物及并萘衍生的化合物的统称，可以抑制并杀灭多种革兰氏阳性菌、革兰氏阴性菌、螺旋体、立克次体及支原体等微生物，因此被称为广谱抗生素^［1，2］^。TCs可分为天然四环素类抗生素和半合成四环素类抗生素两种主要类型，天然四环素类抗生素包括金霉素（chlortetracycline）、四环素（tetracycline）和土霉素（oxytetracycline）。人们对天然四环素类抗生素进行广泛的结构修饰，开发出半合成四环素类抗生素，如强力霉素（doxycycline）、二甲胺四环素（minocycline）、甲烯土霉素（methacycline）和去甲基金霉素（demeclocycline）等^［3］^。TCs因抗菌范围广和使用经济性等优势，广泛应用于我国畜禽及水产养殖业。然而因其滥用带来的耐药性、药物残留和生态破坏等问题也不容忽视^［4-6］^。不当或过量使用TCs会导致其在动物体内残留，并通过食物链进入人体，对人类健康造成潜在威胁。我国GB 31650-2019中规定了四环素、土霉素、金霉素和强力霉素在动物性食品中的最大残留限量（MRL），动物性食品的肌肉中，土霉素、金霉素和四环素的MRL均为200 μg/kg，强力霉素的MRL为100 μg/kg^［7］^。TCs在养殖动物体内和环境中经降解代谢后，会生成异构体和脱水产物，如差向四环素（4-epitetracycline）、差向金霉素（4-epichlortetracycline）和差向土霉素（4-epioxytetracycline）等，其毒性较母体化合物更强，国内已经开展了对四环素类药物原型及其降解产物的兽药残留研究^［8-10］^。

常见的TCs残留检测技术主要包括微生物抑制法^［11，12］^、酶联免疫吸附法（ELISA）^［13］^、高效液相色谱法（HPLC）^［14-16］^和超高效液相色谱-串联质谱法（UPLC-MS/MS）^［17-20］^等。微生物抑制法基于抗生素对微生物生长的抑制效应进行检测，适用于初步筛查和常规监测，但存在灵敏度较低、特异性较差、检测周期较长等局限性，且无法区分TCs的具体种类。ELISA操作简便，分析快速，适合大批量样品的快速筛查和现场检测，但可能存在假阳性和假阴性问题。HPLC具有灵敏度高、准确性好等优点，是TCs残留检测的常规方法，但对样品前处理要求较严格，易受杂质干扰，且分析耗时较长。UPLC-MS/MS兼具高灵敏度和高特异性^［21-23］^，可同时快速检测多种抗生素残留，适用于动物组织、环境样本等复杂基质，现已经成为兽药残留分析的主流技术。

传统的兽药残留前处理检测技术如液液萃取法^［24］^、QuEChERS法^［25］^、固相萃取法（SPE）等，因需繁复的检测前处理步骤，直接影响整个分析实验的效率和可行性。传统SPE填料种类有限，难以实现复杂基质中多种兽药残留的同步检测。张永芳等^［26］^基于SPE和UPLC-MS/MS技术，使用HLB固相萃取柱净化畜禽肉样品，测定了四环素类抗生素（四环素、土霉素、金霉素、强力霉素）的残留量。但这类方法存在检测TCs种类较少、前处理步骤复杂等不足。目前Oasis PRiME HLB作为一种新型SPE吸附剂，采用独特的双模式吸附机制，结合了反相和离子交换作用，能够有效吸附和分离多种类型的兽药残留物，且提取后的样品溶液可直接过柱，无需活化与平衡步骤，极大地提升了检验效率。本研究优化了样品前处理方法、色谱和质谱条件，并对四环素类抗生素标准溶液的存储容器进行了研究。所建立的UPLC-MS/MS方法通过简化的样品净化步骤，实现了动物肌肉组织中多种不同结构形态TCs的同步检测。该方法操作简便，效率高，可准确测定动物肌肉组织中的TCs残留量。

## 1 实验部分

### 1.1 仪器、试剂与材料

I-Class Xevo TQ-S超高效液相色谱-串联质谱仪（美国沃特世公司）；Eclipse Plus C18色谱柱（100 mm×2.1 mm，3.5 μm，美国安捷伦公司）；CT18RT高速冷冻离心机（上海精科天美公司）；N-EVAP氮吹仪（美国Organomation公司）；BSA223S-CW电子天平（感量0.001 g，德国赛多利斯公司）；Multi Reax型多管涡旋振荡器（德国Heidolph公司）。

TCs标准物质购自天津阿尔塔科技有限公司，纯度均大于98%。

Oasis PRiME HLB固相萃取柱（6 mL/200 mg）、2 mL透明液相色谱进样瓶（美国沃特世公司）；甲酸、甲醇、乙腈（色谱纯，德国Merck公司）；0.22 μm尼龙针式过滤器（天津博纳艾杰尔科技有限公司）；猪肉、鸡肉和鱼肉样品（山东省烟台市农贸市场）。

### 1.2 标准溶液的配制

分别准确称取适量TCs标准物质，用甲醇溶解配制成100 μg/mL的标准储备液，于-18 ℃冰箱保存。分别移取适量标准储备液，用甲醇配制成1 μg/mL的混合标准中间液，于-18 ℃冰箱保存。根据实验需要，移取适量混合标准中间液，用空白基质溶液配制成质量浓度为1、5、10、50、100、200、500 ng/mL的混合基质标准溶液。

### 1.3 样品前处理

提取：称取均质后试样2 g（精确至0.001 g），加入10 mL含0.2%甲酸的80%乙腈水溶液，涡旋混匀，振荡提取15 min，5 ℃下10 000 r/min离心5 min，上清液待SPE净化。

净化：取6 mL待净化液于Oasis PRiME HLB固相萃取柱，重力过柱。移取5.00 mL流出液至氮吹管，40 ℃氮吹至近干。用50%甲醇水溶液定容至1 mL，超声溶解。复溶液经滤膜过滤后转移至进样瓶，立即UPLC-MS/MS测定。需同时进行空白试验。

### 1.4 分析条件

#### 1.4.1 色谱条件

Eclipse Plus C_18_色谱柱（100 mm×2.1 mm，3.5 μm）；柱温40 ℃；进样量5 µL；流速0.2 mL/min；流动相A：0.1%甲酸水溶液，流动相B：0.1%甲酸乙腈溶液，梯度洗脱：0～2.0 min，5%B；2.0～3.5 min，5%B～15%B；3.5～7.0 min，15%B～20%B；7.0～9.0 min，20%B～65%B；9.0～9.1 min，65%B～90%B；9.1～10.0 min，90%B；10.0～10.1 min，90%B～5%B；10.1～12 min，5%B。

#### 1.4.2 质谱条件

电喷雾电离正离子（ESI^+^）模式扫描，多反应监测（MRM）采集模式；毛细管电压0.5 kV；离子源温度150 ℃；脱溶剂气温度300 ℃；脱溶剂气流速800 L/h；锥孔气流速150 L/h；锥孔电压：30 V；碰撞气流速0.15 mL/min，其他质谱参数见表1。

**表 1:** 10种四环素类抗生素的保留时间和质谱参数

Compound	*t*_R_/min	Parent ion （*m/z*）	Daughter ions （*m/z*）	Collision energies/V
Minocycline	5.24	458.2	441.1^*^/352.1	20/30
4-Epioxytetracycline	5.99	461.2	444.2^*^/426.2	15/20
4-Epitetracycline	6.04	445.0	410.0^*^/427.4	20/13
Oxytetracycline	6.19	461.2	442.9^*^/426.2	14/20
Tetracycline	7.05	445.3	410.0^*^/427.3	20/13
Demeclocycline	8.39	465.0	448.0^*^/430.0	16/20
4-Epichlortetracycline	8.21	479.1	462.2^*^/444.2	15/20
Chlortetracycline	9.10	479.3	444.2^*^/462.2	20/15
Methacycline	9.14	443.0	426.0^*^/381.0	15/20
Doxycycline	9.23	445.2	428.2^*^/154.0	15/30

* Quantitative ion.

## 2 结果与分析

### 2.1 质谱条件的优化

在正离子和负离子模式下，分别使用流动注射方式将200 ng/mL单个化合物溶液注入质谱仪。目标物在正离子模式下进行离子化可达到最优的灵敏度，故在ESI^+^模式下对*m/z* 100~500范围内的离子进行全扫描，确定每一个目标物质的母离子，再进行子离子扫描，每个目标物质选取2对响应值高的特征离子对分别作为定量、定性离子对，再进一步对碰撞能量进行优化，优化结果见表1。

### 2.2 色谱条件的优化

#### 2.2.1 流动相的选择

为了优化TCs电离效果和峰形，考虑到在进行质谱分析时，使用正离子模式，TCs在软电离过程中需要捕获H^+^，分别向水-乙腈和水-甲醇的流动相体系中添加0.1%甲酸、5 mmol/L乙酸铵缓冲盐和5 mmol/L甲酸铵缓冲盐。结果显示：在流动相体系中分别添加5 mmol/L乙酸铵缓冲盐和5 mmol/L甲酸铵缓冲盐时，目标化合物的色谱峰形得到一定改善，但响应降低较大。在流动相体系中添加0.1%甲酸后，目标化合物的峰形和分离效果得到明显改善，化合物响应提高。对比0.1%甲酸水溶液-0.1%甲酸甲醇溶液体系和0.1%甲酸水溶液-0.1%甲酸乙腈溶液体系，结果发现，有机相为乙腈时大多数TCs的峰形和响应优于甲醇体系，故选择0.1%甲酸水溶液-0.1%甲酸乙腈溶液作为流动相。

#### 2.2.2 色谱柱的选择

为了进一步优化TCs（特别是结构相似的同分异构体）的色谱分离效果，本研究对比了Eclipse Plus C_18_（100 mm×2.1 mm，3.5 μm）和BEH C_18_（100 mm×2.1 mm，1.7 μm）两种C_18_色谱柱的分离效果。实验结果显示：对于结构相似的同分异构体，使用Eclipse Plus C_18_色谱柱时TCs可以得到良好的峰形和分离效果。以差向四环素和四环素为例，使用Eclipse Plus C_18_色谱柱，二者可达到完全基线分离，且保留时间相差1 min以上；而使用BEH C_18_色谱柱，两者无法达到良好的分离效果，且保留时间相差仅0.2 min，影响定量和定性分析（见图1）。因此选择Eclipse Plus C_18_色谱柱作为实验用色谱柱。图2为四环素类抗生素（200 ng/mL）的提取离子流色谱图。

**图1:**
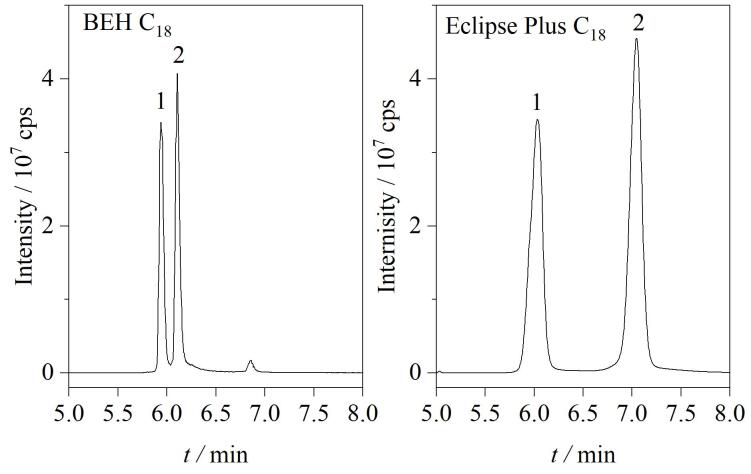
采用不同色谱柱时四环素和差向四环素的提取离子流色谱图

**图2:**
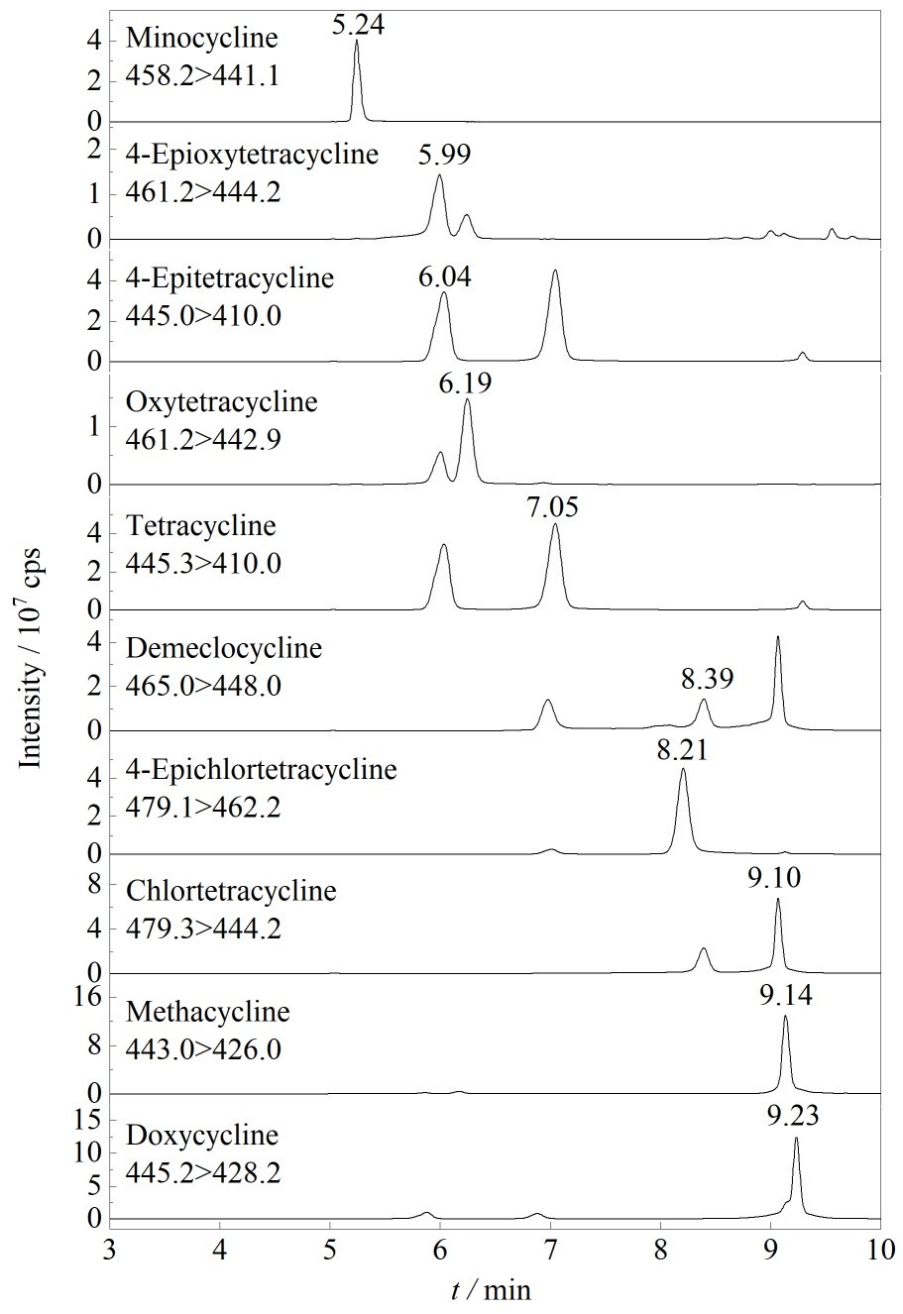
10种四环素类抗生素的提取离子流色谱图

### 2.3 前处理方法的优化

#### 2.3.1 提取溶剂的选择

在对动物肌肉组织中的兽药残留进行同步检测时，选用恰当的提取溶剂至关重要。在选择提取溶剂时，需考虑目标物在溶剂中的溶解度以及溶剂对基质的浸透性等关键因素。乙腈作为一种极性溶剂，作为提取溶剂时，能够有效防止样品中脂肪的过多提取，并且对蛋白质的沉淀效果显著。TCs含有多个亲水羟基，易溶于水，在中性和酸性环境下表现出较高的稳定性^［27］^。通过调整水相和甲酸的比例，可以改变乙腈的极性和酸度，进而优化多种目标化合物的提取效率。实验研究了纯乙腈、不同比例（9∶1、8∶2、7∶3、6∶4，体积比）乙腈-水和分别添加不同体积分数（0.1%、0.2%、0.5%）甲酸作为样品提取溶剂对样品蛋白质的沉淀效果和目标物的提取率，计算了TCs的平均回收率，见图3。使用纯乙腈作为样品提取液会导致试样凝聚成团，不利于试样的均匀分散，TCs回收率均低于70%。随着乙腈比例的降低，回收率有所改善，但过高的水相比例会提取更多干扰杂质。随着甲酸比例升高，对蛋白质的沉淀除杂效果显著增强，但平均回收率低于65%。为了提高TCs的整体回收率并保持其稳定性，综合回收率等因素，选择含0.2%甲酸的乙腈-水（8∶2，体积比）混合溶液作为样品提取溶剂（平均回收率为75%）。

**图3:**
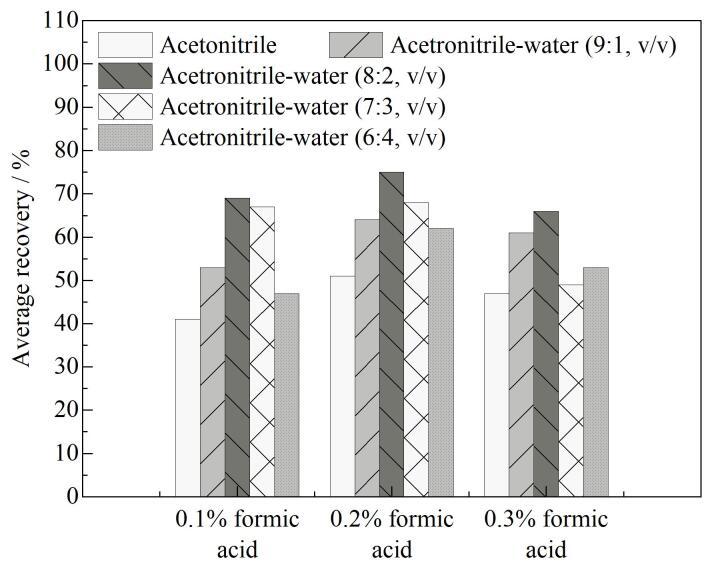
使用不同提取溶剂时四环素类抗生素的平均回收率

#### 2.3.2 固相萃取柱的选择

兽药残留因样品基质复杂，常采用SPE来消除基质干扰。区别于单一填料的传统HLB固相萃取柱，新型Oasis PRiME HLB固相萃取柱对酸性、碱性化合物的选择性不受溶液酸碱性影响，且对各种化合物的吸附效果较为接近。相较于使用HLB固相萃取柱需活化、平衡、淋洗、洗脱等步骤，使用Oasis PRiME HLB固相萃取柱仅通过简易的一步式净化，即可消除常见的蛋白质和磷脂等基质干扰，并显著提高实验效率。本研究通过加标回收试验，对比了上述两种固相萃取柱的回收率。选用Oasis PRiME HLB固相萃取柱时TCs回收率结果（62.6%~119.0%）略优于HLB固相萃取柱（54.2%～89.3%）。综合考虑前处理实验效率和回收率结果，选择Oasis PRiME HLB固相萃取柱作为实验用净化柱。

#### 2.3.3 样品瓶的选择

因TCs与玻璃包装可能发生相互作用，主要涉及药物吸附、降解等问题^［28］^。《中华人民共和国药典》（2020年版）根据药用玻璃材料颗粒耐水性的高低，将药用包装玻璃分为3类。Ⅰ类玻璃为硼硅类玻璃，具有高耐水性；Ⅱ类玻璃为钠钙类玻璃内表面经过中性化处理后的玻璃，具有较高耐水性；Ⅲ类玻璃为钠钙类玻璃，具有中等耐水性。当药物有避光要求时，可选择棕色玻璃。本实验选取了上述3类玻璃材料样品瓶（type Ⅰ、Ⅱ、Ⅲ）和对应的3类棕色玻璃材料样品瓶（amber type Ⅰ、Ⅱ、Ⅲ），研究了实验过程中配制的TCs标准溶液在6类不同玻璃材料样品瓶中的变化情况。

实验用TCs混合溶液（100 ng/mL）模拟TCs标准储备液的存储情况。研究中分别将TCs混合溶液注入6类不同玻璃材料的样品瓶中，-18 ℃避光存储。10天内每天固定时间分别测定存储混合溶液的样品瓶和当天注入新配制混合溶液的样品瓶中TCs的响应值，分别用两者的响应强度比值作为纵坐标，实验天数作为横坐标，记录6类不同玻璃材料样品瓶中TCs的变化，见图4。

**图4:**
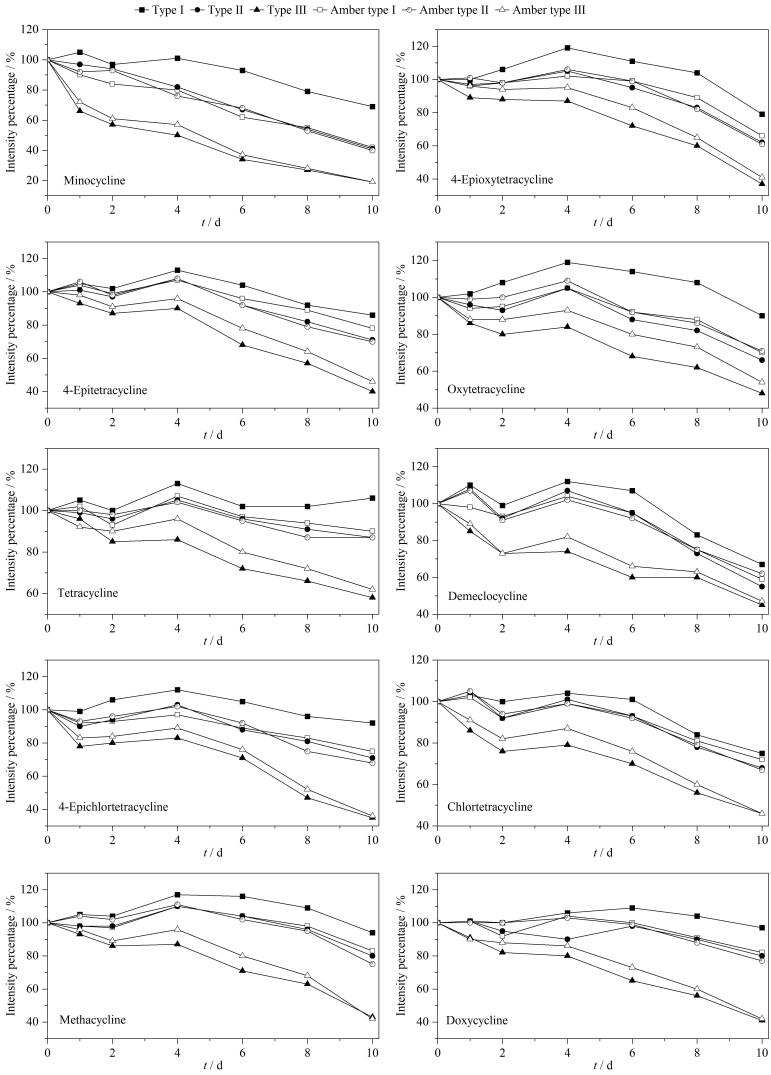
6类不同玻璃材料样品瓶中四环素类抗生素的响应变化

从图4可见，在10天存储期内，二甲胺四环素、差向四环素、土霉素、四环素和去甲基金霉素在Ⅰ类玻璃中变化最小，在棕色Ⅰ类玻璃、Ⅱ类玻璃和棕色Ⅱ类玻璃变化较小，在Ⅲ类玻璃和棕色Ⅲ类玻璃中变化较大；其余TCs在Ⅲ类玻璃和棕色Ⅲ类玻璃中变化较大，在其他4种玻璃材料中变化都较小。

实验结果表明，使用Ⅰ类玻璃材料存储TCs标准溶液，具有较好的化学稳定性，Ⅱ类玻璃和棕色Ⅰ类、Ⅱ类玻璃材料次之，Ⅲ类玻璃和棕色Ⅲ类玻璃材料较差，因此，实验选择Ⅰ类玻璃材料样品瓶存储TCs标准溶液。

### 2.4 方法学验证

#### 2.4.1 基质效应（ME）

ME是质谱检测中普遍存在的现象，通常采用基质标准曲线斜率与溶剂标准曲线斜率的百分比来评价基质效应。本研究按照韩林学等^［29］^的方法，分别考察了猪肉、鸡肉和鱼肉的基质效应。按照本文1.3节进行空白基质前处理，得到空白基质溶液后，绘制基质标准曲线，同时使用50%甲醇水溶液绘制相同浓度的试剂标准曲线。以两条标准曲线斜率的比值来评估ME。当ME为80%～120%时，判断为弱基质效应，当ME为50%～80%和120%～150%时，判断为中基质效应，当ME为小于50%或大于150%时，判断为强基质效应。结果表明，3种基质中TCs大多表现为中和弱基质效应。因此，为降低基质抑制对化合物的影响，采用空白基质标准曲线进行定量检测。

#### 2.4.2 线性关系、检出限与定量限

用动物肌肉组织（猪肉、鸡肉和鱼肉）的阴性基质提取液配制质量浓度为1~500 ng/mL的基质混合标准工作溶液，在优化条件下进行测定，根据色谱峰面积与分析物的质量浓度建立基质标准曲线，TCs在质量浓度范围内线性关系良好，相关系数（*r* ^2^）均大于0.994。符合GB/T 27404-2008中*r* ^2^不应低于0.99的要求。在阴性基质中分别加入低浓度的混合标准溶液，按1.3节方法处理后测定，以信噪比（*S/N*）=3确定方法的检出限（LOD），以*S/N*=10确定方法的定量限（LOQ）。TCs在3种基质中的LOD为0.10～0.15 μg/kg，LOQ为0.20～0.50 μg/kg（见表2）。

**表 2:** 四环素类抗生素检出限和定量限

Compound	Pork		Chicken		Fish meat	
	LOD/（μg/kg）	LOQ/（μg/kg）	LOD/（μg/kg）	LOQ/（μg/kg）	LOD/（μg/kg）	LOQ/（μg/kg）
Minocycline	0.15	0.40	0.15	0.50	0.15	0.50
4-Epioxytetracycline	0.10	0.30	0.15	0.50	0.15	0.50
4-Epitetracycline	0.15	0.50	0.15	0.50	0.15	0.50
Oxytetracycline	0.10	0.30	0.15	0.40	0.10	0.20
Tetracycline	0.15	0.50	0.15	0.50	0.10	0.20
Demeclocycline	0.15	0.50	0.15	0.40	0.15	0.40
4-Epichlortetracycline	0.15	0.50	0.10	0.30	0.10	0.20
Chlortetracycline	0.15	0.40	0.15	0.50	0.15	0.50
Methacycline	0.10	0.20	0.15	0.40	0.15	0.40
Doxycycline	0.10	0.20	0.10	0.30	0.10	0.20

#### 2.4.3 回收率与相对标准偏差

在动物肌肉组织（猪肉、鸡肉和鱼肉）的阴性基质试样中，进行低（1 μg/kg）、中（5 μg/kg）、高（10 μg/kg）3个水平的加标回收试验，每个水平进行7次平行试验，计算各待测TCs的平均回收率和相对标准偏差（RSD）。结果显示TCs在3种基质的平均回收率为62.6%～119.0%，RSD为2.0%～9.8%（见表3），符合GB/T 27404-2008中回收率和精密度的要求。其中甲烯土霉素的回收率略低，考虑其结构中多一个甲烯基，增大了其疏水性，在基质中钙离子易与甲烯土霉素结合。此外，本研究还发现，在不同种类的动物肌肉组织样本中，TCs的回收率和精密度呈现出一定的差异。这可能与基质中脂肪含量、蛋白质结构以及水分含量等多种因素相关。

**表 3:** TCs在猪肉、鱼肉、鸡肉中的平均加标回收率和相对标准偏差（*n*=7）

Compound	Added/ （μg/kg）	Pork		Chicken		Fish meat	
		Recovery/%	RSD/%	Recovery/%	RSD/%	Recovery/%	RSD/%
Minocycline	1	119.0	6.5	81.0	8.1	106.3	9.1
	5	118.3	9.6	78.3	6.7	98.3	8.6
	10	113.5	4.0	84.9	4.8	88.4	8.9
4-Epioxytetracycline	1	94.8	8.6	80.3	7.5	108.5	3.5
	5	102.0	3.0	89.4	8.9	100.5	8.8
	10	77.9	3.2	71.6	4.3	86.3	3.4
4-Epitetracycline	1	86.3	4.9	65.0	8.7	116.8	5.1
	5	100.4	4.5	75.6	7.2	96.5	8.9
	10	70.3	4.9	73.6	5.8	89.5	4.8
Oxytetracycline	1	91.5	3.5	73.0	8.9	107.5	3.0
	5	104.3	3.1	84.4	7.3	93.8	8.9
	10	82.2	2.3	76.9	3.2	72.1	4.2
Tetracycline	1	117.0	3.2	97.0	9.7	116.5	3.9
	5	115.9	3.7	110.1	9.5	110.9	9.3
	10	88.1	4.7	84.0	3.2	84.1	3.8
Demeclocycline	1	65.5	5.8	63.5	9.8	104.5	2.0
	5	77.5	4.0	70.0	7.7	103.8	8.0
	10	70.7	4.5	63.7	3.8	81.4	2.7
4-Epichlortetracycline	1	69.8	9.6	69.8	7.7	86.8	3.3
	5	83.5	3.9	74.3	9.4	80.0	9.5
	10	62.8	3.3	65.1	3.5	78.8	5.4
Chlortetracycline	1	71.0	4.2	66.0	4.6	77.3	2.3
	5	77.1	3.3	68.4	9.2	74.5	9.3
	10	64.8	3.2	64.7	4.6	69.2	2.5
Methacycline	1	65.3	7.0	67.5	6.0	65.0	4.3
	5	75.1	4.1	62.7	5.1	67.6	8.5
	10	62.6	2.3	64.0	4.9	65.5	9.8
Doxycycline	1	79.0	9.0	73.3	3.9	75.3	4.4
	5	87.1	5.3	76.1	5.1	70.1	9.3
	10	73.3	3.3	68.6	4.3	66.2	2.5

### 2.5 方法实际应用结果

按照上述实验方法，在辖区农贸市场内抽取猪肉、鸡肉和鱼肉样品各20份进行残留量的测定。结果显示：在2份猪肉样品中检出四环素残留（2.3和5.2 μg/kg）；在3份鸡肉样品中检出强力霉素残留（3.6、4.1和4.6 μg/kg）；在1份鱼肉样品中检出土霉素残留（2.1 μg/kg）。所有检出样品的残留量均低于国家限量标准值（200 μg/kg）。TCs在猪肉、鸡肉和鱼肉样品的检出率分别为10%、15%和5%，说明该类药物在养殖过程中使用较频繁，需监控用药和残留的情况。

## 3 结论

本实验建立了一种快速测定动物肌肉组织样品中TCs含量的分析方法，优化了前处理技术和检测仪器条件，具有前处理简单、检出限和定量限低、回收率较高和重复性好等优点。实验采用Oasis PRiME HLB固相萃取柱对提取液进行一步式简易净化除杂，显著提高了前处理效率。通过对液相色谱和质谱参数的优化，实现了对动物肌肉组织中四环素类抗生素残留的快速定性定量检测。本方法简单、快速，灵敏可靠，为快速准确测定动物肌肉组织中TCs提供了更多的可行性研究。此外，该方法不仅适用于动物肌肉组织样品，还具有一定的通用性，可拓展至其他生物样品（如鸡蛋、肝脏等）的TCs残留分析。未来研究可以进一步探索该方法在不同基质中的适用性，为食品安全监管提供更加高效、准确的检测手段。
